# Poultry gut health – microbiome functions, environmental impacts, microbiome engineering and advancements in characterization technologies

**DOI:** 10.1186/s40104-021-00640-9

**Published:** 2021-12-02

**Authors:** Christiana Eleojo Aruwa, Charlene Pillay, Martin M. Nyaga, Saheed Sabiu

**Affiliations:** 1 Department of Biotechnology and Food Science, Faculty of Applied Sciences, Durban University of Technology, P.O. Box 1334, Durban, 4000 South Africa; 2 Next Generation Sequencing Unit, Division of Virology, Faculty of Heath Sciences, University of the Free State, Bloemfontein, South Africa

**Keywords:** Disease, Environmental impacts, Gut microbiome, Microbiome engineering, Poultry health

## Abstract

The gastrointestinal tract (GIT) health impacts animal productivity. The poultry microbiome has functions which range from protection against pathogens and nutrients production, to host immune system maturation. Fluctuations in the microbiome have also been linked to prevailing environmental conditions. Healthy poultry birds possess a natural resistance to infection. However, the exploration of environmental impacts and other relevant factors on poultry growth and health have been underplayed. Since good performance and growth rate are central to animal production, the host-microbiome relationship remains integral. Prior to the emergence of metagenomic techniques, conventional methods for poultry microbiome studies were used and were low-throughput and associated with insufficient genomic data and high cost of sequencing. Fortunately, the advent of high-throughput sequencing platforms have circumvented some of these shortfalls and paved the way for increased studies on the poultry gut microbiome diversity and functions. Here, we give an up-to-date review on the impact of varied environments on microbiome profile, as well as microbiome engineering and microbiome technology advancements. It is hoped that this paper will provide invaluable information that could guide and inspire further studies on the lingering pertinent questions about the poultry microbiome.

## Introduction

The microbiome refers to the totality of microoganisms associated with an organism [1]. The microbiome has complex interactions with their hosts which could be harmful (pathogenic) or beneficial (symbiotic) and can play key roles in human and animal health. Many members of the microbiome are however non-culturable and require more sensitive methods for their identification and enumeration. For example, more than 70% of human gut bacteria cannot be cultured on common laboratory media [2]. Nonetheless, given the advancements in molecular biology and emergence of high-throughput molecular sequencing technologies that are culture-independent, new and interesting scientific data continue to emerge and shed light on microbiome interactions in their host(s) [1].

High-throughput sequencing methods in host (poultry)-gut microbiome analysis may involve the use of specific markers in amplicon sequencing or metagenomic approaches [3]. These analytical approaches may or may not be target-oriented. In target-oriented techniques genes shared by members of a microbiome become the subject of analysis. The gene fragments amplified then yield sequence reads that are representative of the genetic pool and diversity in the sample population under study. So, genetic variants within study samples are reflected in the sequence reads abundance. From the amplified reads, phylogenetic information can be derived, as exemplified in the highly elaborated 16S ribosomal ribonucleic acid (RNA) gene, which serves as an excellent tool for microbiome analysis [4]. Thereafter, microbial taxons are characterized through de novo clustering of targeted sequence regions or by comparing derived sequence reads with reference sequences from relevant databases. Amplicon or whole shotgun sequencing has the advantage of allowing the detailed analysis of an entire microbiome while being less affected by sample size [1].

Poultry gut health impacts on poultry productivity and is an integral subject worthy of scientific research [5]. In other words, adverse effects on the gut health could partially or wholly affect poultry health and impede nutrient uptake and utilization. Consequently, gut health involves a complex network of interactions, including its structural integrity on a larger and microscale. Gut health also involves microbiome balance and impacts on immunity status of the host. Accumulating evidence suggests a strong impact of gut health on poultry productivity [6].

Poultry products are high in protein and fatty acids [7]. Poultry implies domesticated birds, for example, turkey, pigeons, guinea fowls, geese, duck, squab, quails and chickens, which are usually kept for their feathers, eggs or meat. They are typical members of the order Galliformes and may include game birds, land and waterfowls [7]. Chickens are more domesticated and widely reared for meat [8]. However, increased urbanization trends, world populations and meat demands have driven bulk production practices through more intensive and specialised poultry farming units. This is directed towards providing safe and cheap poultry, and poultry products [9]. Whether poultry farming occurs on a subsistent or commercial scale, animal welfare concerns need to be addressed at an early stage to ensure optimal production and profits to farmers. For instance, poultry farmers must be aware that diseases could spread from one poultry flock to another and can be caused by a variety of factors such as changes in feed, weather and environmental conditions which impact poultry gut health [10]. Again, the lack of preventive or control measures in poultry farming systems have been linked to numerous disease outbreaks of avian origin and culminated in huge economic loss [11].

Poultry health for sustained meat supply is closely linked to their gut microbiome profile and diversity. Microbiome functions include protection against pathogens, nutrients production, and host immune system maturation [12, 13]. The presence of a healthy and functional gut microbiome is essential to poultry performance and health. Following the ban in European, American and some African countries on the use of antimicrobials to promote poultry growth and fight infections, there has been an increase in poultry digestive diseases due to dysbiosis, that is, imbalance in gut microbiome. A poor gastrointestinal (GI) health may result in nutrients malabsorption and attendant growth depression in affected poultry birds [14]. The changes in farming practices and environments may also impact microbiome profile by influencing poultry natural immunity [15]. Age of poultry also affects their gut microbiome diversity [16]. Healthy poultry birds possess an innate resistance to infections [17], but the host-microbiome relationship remain important for good poultry performance and growth [15]. Poultry birds that are most domesticated and studied include the chicken (*Gallus* spp.) and turkey (*Meleagris* spp.). Other less domesticated birds include the duck (*Carina* spp.) and geese (*Anser* spp.) [8]. This article is therefore targeted towards giving an up-to-date account of the poultry gut microbiome, their functions, impacts of varied environmental conditions, means of gut microbiome engineering, developments in microbiome technologies and prospects.

## Materials and methods

**Fig. 1:**
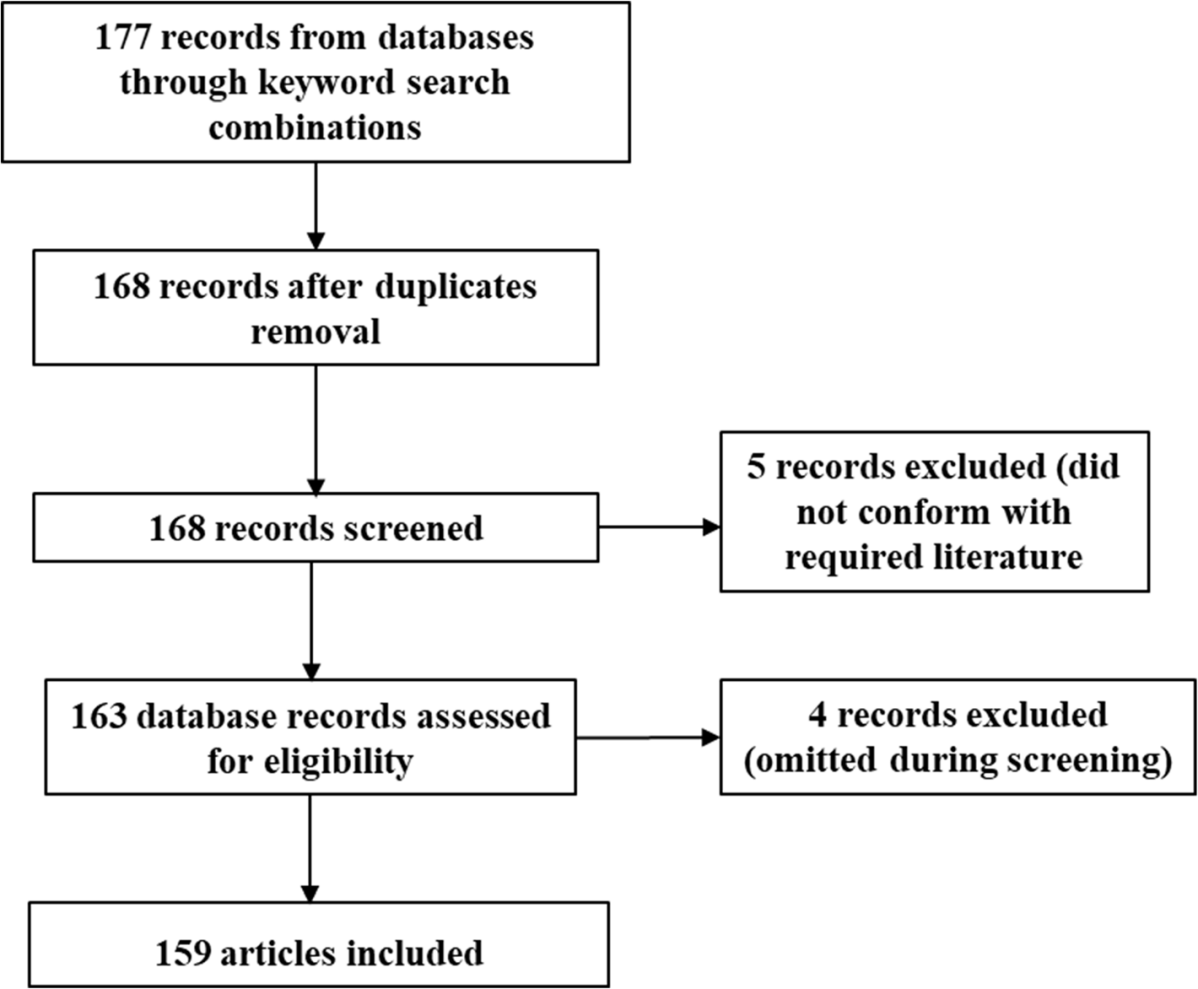
PRISMA flow chart showing number of recognized search records, screened, included and excluded materials used in this review

The data and information in this article were retrieved from sources such as PubMed, ScienceDirect, Scopus, MeSH, Food and Agricultural Organization (FAO) websites and other reputable online scientific databases. Keywords and phrase combinations which were considered relevant to the topic of interest and scope include host-microbe interaction, poultry microbiome, microbiome functions and mechanisms, poultry classification, the gastrointestinal tract microbiome, microbiome engineering, microbiome role in poultry health, microbiome and poultry conservation, and factors affecting the gut microbiome. Records used in this review covered from 1973 to April 2021. This was done to refine search results for up-to-date objective assessment and report on the poultry microbiome, their impact on health and to provide perspectives that could guide future studies in poultry microbiome. The data collected were used to create a PRISMA flow chart (Fig. 1) depicting records used and their screening process.

## The poultry microbiome

### The chicken microbiome (bacteria)

Generally, the dominant bacteria phyla reported in chickens include the Proteobacteria, Firmicutes, Actinobacteria and Bacteroidetes [18]. About 31 genera within the Firmicutes family with ≥ 5% representing the *Eubacterium*, *Ruminococcus* and *Clostridium* have been reported. The other genera identified from sequencing were the *Riemeralla*, *Paraprevotella*, *Tanneralla* and *Prevotella* [19]. Bacteroidetes constituted 40% of the general microbiome. Predominant Proteobacteria genera included *Neissenia*, *Desulfohalobium*, *Shigella* and *Escherichia* [19]. Within the poultry caecal sacs, there are high concentrations of bacteria present (10^10^–10^11^ cells per gram cecal material) that encode for greater than 95% of the genetic information [20]. Many of the bacteria present in the chicken caecum have remained unculturable in the laboratory and can only be identified through high-throughput sequencing techniques [21].

The dominant microbiota present in the ileum of chickens are the facultative and microaerophilic bacteria, lactobacilli [22]. There are over 40 different types of anaerobic Gram-negative and Gram-positive, non-spore-forming rods and cocci; and several clostridial species identified in the chicken caeca by culturable approaches [23]. The analyses of caecal microbiota by molecular approaches have identified bacterial populations of over 600 species from more than 100 genera. However, many of these bacteria remain as unclassified species or genera [9, 19, 24]. A varied composition of bacteria between the jejunum and the caecum have been reported. *Acinetobacter* and *Acidobacteria* dominate the jejunal microbiota, while *Bacteroides* and *Clostridium* are the predominant bacteria within the caecum [25].

The chicken intestinal microbiome commonly contains several taxa. Non-pathogenic *Campylobacter* spp. such as *Campylobacter jejuni* and *C. coli* may be present in concentrations up to 10^7^ colony forming unit per gram (cfu/g) in the chicken intestine and can be cultured from a week-old chick [26]. *Salmonella* was detected in lower concentrations in the intestinal microbiome and is distributed sporadically [27]. *Salmonella* is pathogenic in avians; however, disease susceptibilities are dependent on host age [28], health status of the immune system [29], and strain type of *Salmonella* [30]. Other bacteria present in the GIT of chickens at lower concentrations are *Escherichia coli*. Certain strains of *E. coli* may however cause opportunistic secondary infections in poultry birds [31]. The increased levels of ammonia present in poultry housing environments can promote such infections [32]. Many avian pathogenic *E. coli* (APEC) isolates known to possess virulence genes like the P-pili, Ibe proteins or K1 capsule are phylogenetically related to human extra-intestinal *E. coli* pathotypes [33]. However, these virulent factors may be found to be distributed sporadically in many avians [34].

### Bacterial succession and transmission in chickens

The gut is colonized by microorganisms shortly after hatching [35]. Immediately after hatching, the GIT of poultry birds encounters many exogenous microorganisms. The initial colonizers and microbiota provide a baseline environment for the creation of a stable and divergent population over time [35, 36]. Initially, the gut of chickens is colonized by facultative aerobes and later substituted by anaerobes [25]. The prolific growth and oxygen consumption by aerobic bacteria create reducing conditions in the gut ecosystem which promote the subsequent growth and colonization by obligate anaerobes [37].

The developmental growth and aging of poultry initiate successional changes in the GIT microbiome composition such that the microbiome profile diversifies till it reaches a stable state [38]. The first bacteria detected to colonize the caeca within the first hour of life are the streptococci and enterobacteria which are then distributed throughout the intestinal tract within 24 h. On day three, many more bacteria such as lactobacilli, streptococci, enterococci and coliforms can be isolated from the different parts of the GIT [39]. The diverse bacterial population found in the chicken caecum increases during the first 6 weeks of life. At the age of 3 weeks, the bacterial population of chickens shift from Proteobacteria, *Bacteroides* and Firmicutes to only Firmicutes [40]. In a study conducted by Awad et al. [41], a high bacterial population was observed from jejunal and caecal mucosal samples from day-old chickens. This was indicative of an increased intake of microorganisms from the surrounding environment after hatching. The composition of the microbiome found in the gut of avians differed between the younger and older birds. Proteobacteria were found at increased levels during the first fews days of life and thereafter decreased. However, in older birds, Firmicutes such as Lachnospiraceae, Ruminococcaceae, Clostridiaceae, and Lactobacillaceae were the most dominant phyla present [25]. In a study conducted by Lu et al. [42], *Lactobacillus delbrueckii*, *Clostridium perfringens* and *Campylobacter coli* were detected in the GIT of chickens at 3 days of age, whereas *L. acidophilus*, *Enterococcus* and *Streptococcus* was found in chickens from 7 to 21 days of age [42]. The caecal microbiota found in two-week-old chickens comprised of Gram-positive (anaerobic cocci, *Eubacterium* spp., *Lactobacillus* spp., *Clostridium* spp.) and Gram-negative (*Fusobacterium* spp. and *Bacteroides* spp.) anaerobes [43]. When chickens were more than 40 days old, the GIT comprised of Gram-positive cocci, *Bifidobacterium* spp., *Clostridium* spp. *Streptococcus* spp., *E. coli*, *Bacteroides* spp. and *Lactobacillus* spp. [39].

In the case of mother hen-to-offspring microbiome transmission, the phyla Bacteroidetes (*Bacteroides, Prevotella*, *Paraprevotella*, *Hallella*, *Butyricimonas*, *Tannerella*, *Alistipes*, *Rikenella*, *Parabacteroides*, *Barnesiella*, *Odoribacter* and *Phocaeicola* genera); Proteobacteria (*Desulfovibrio*, *Bilophila*, *Anaerobiospirillum*, *Succinatimonas*, *Succinivibrio*, *Helicobacter*, *Campylobacter*, *Sutterella*, *Parasutterella*); Deferribacteres (*Mucispirillum*) and Firmicutes (*Phascolarctobacterium*, *Megamonas*, *Megasphaera*, *Dialister*, *Erysipelotrichaceae incertae sedis*, *Dorea*, *Acetitomaculum*, *Faecalibacterium*, *Subdoligranulum*, *Gemmiger*, *Peptococcus*, *Eubacterium*, *Guggenheimella*, *Defluviitalea*) constituted the major microbiome genera [44]. Other less dominant phyla and genera include the Synergistetes (*Cloacibacillus*), Actinobacteria (*Olsenella*, *Collinsella*, *Bifidobacterium*), Spirochaetes (*Treponema*, *Spirochaeta*), Tenericutes (*Asteroleplasma*) and *Candidatus saccharibacteria* (*Saccharibacteria incertae sedis*) [44]. In another scenario, a more diverse microbiome was demonstrated in high egg-laying chickens compared to low egg-laying hens. The former had *Lactobacillus*, *Bifidobacterium*, *Acinetobacter*, Flavobacteriaceae, *Lachnoclostridum* and *Rhodococcus* present with relative abundance of Firmicutes, *Bacteroides* and *Fusobacteria* in their faecal profile. The latter group of layer hens showed more actinobacteria, proteobacteria and cyanobacteria [45].

### The chicken enteric virome

The viruses that cause poultry diseases may cause damage to the GIT of young poultry and thus create an environment conducive for the growth of harmful bacteria and protozoa [46, 47]. However, more recent research has shifted focus towards community-based analyses of the gut microbiome. Viral gene sequencing and analysis have revealed the identities of Caudovirales and Geminiviridae viruses and a few uncharacterized viruses in the poultry gut [48]. The undetermined roles of these novel viruses in enteric disease syndromes in poultry or in the overall gut health of poultry have still to be characterized and understood. Therefore, it is important to determine the viral constituents of the poultry gut and to identify and characterize these viruses [49]. Interestingly, recent research efforts using electron microscopy, next-generation sequencing (NGS) and rapid diagnostic methods have described novel avian rotaviruses, astroviruses, parvoviruses, picornaviruses and calicivirus in chicken. These are small viruses linked to poultry diseases [50]. Members of the Siphoviridae, Herpesviridae, Retroviridae and Myoviridae viral families have also been recently reported in broiler chickens using NGS [51]. Considering the foregoing, little is known about the poultry virome profile. The difficulty in characterizing members of the poultry virome may be linked to sequencing methods used in virome analyses, as well as the absence of common genes among viruses.

### The turkey microbiome (bacteria)

The composition and role of the bacterial population within the turkey GIT are relatively undetermined [52]. Some studies have compared the caecal microbiome of both wild and domestic birds [53, 54]. These studies have identified specific genera present in the caeca of different types of turkeys, as well as time-dependent shifts in bacterial populations in the turkey intestinal tract [54]. The predominant genera shared by, and found in both chicken and turkey were *Clostridium*, *Ruminococcus*, *Lactobacillus* and *Bacteroides*. Factors such as diet, rearing environment, the digestive passage rate and genetics may contribute to the differences in composition of the intestinal microbiome between the chicken and turkey [36]. The majority of microbiome in turkeys were shown to consist of Gram-positive rods (77%), Gram-negative rods (14%) and Gram-positive cocci (9%). The predominant bacteria isolated were *Eubacterium, Lactobacillus, Peptostreptococcus, Escherichia coli, Propionibacterium* and *Bacteroides* [36]. The dominant phyla found in turkeys of any age and GIT location were the Firmicutes, Bacteroidetes, Actinobacteria and Proteobacteria [55].

In the turkey caeca, Bacteroidetes were found to be more abundant, while the Firmicutes were present in higher concentrations in the small and large intestines [55]. *Alistipes, Bacteroides, Barnesiella, Butyricoccus, Clostridium_XIVb, Hallela, Paraprevotella, Phascolarctobacterium, Pseudoflavonifractor, Roseburia, Ruminococcus, Slackia* and *Syntrophococcus* genera were found in higher abundance in the caeca irrespective of age [36]. In the cloaca, Proteobacteria were the predominant phyla, and their presence was attributed to their oxygen tolerant nature [55]. In the caeca, *Blautia* and *Campylobacter* had higher abundances, while *Anaerovorax* and *Corynebacterium* dominated the cloaca. A significantly lower abundance of *Lactobacillus* and *Streptococcus* was found in the caeca compared to other GIT locations [36]. The age of turkey affects the distribution of the microbiome within the gut. In the gut of 16-week-old turkeys, a higher abundance of Actinobacteria, Bacteroidetes and Proteobacteria were detected. Firmicutes were more predominant in the gut of 6- and 10-week-old turkey birds compared to 16-week-old birds [55].

The analysis of NGS-based data has indicated that the microbiome in the GIT of turkeys is distinctly different from that found in chickens and depicts a 16%–19% similarity at the species level [56]. NGS-based techniques have detected *Campylobacter* spp. as residents of the caeca of turkeys and chickens [55]. A study conducted by Wilkinson et al. [55] determined that both gut location and turkey age may have contributing effects on the microbiome present in the gut. Comparatively, turkey and chicken have been shown to share some genera (*Bacteroides*, *Ruminococcus*, *Clostridium* and *Lactobacillus*), each however showing different distribution pattern. Wei et al. [19] reported a 68% and 89% sequence coverage for microbiome diversity at the species level in turkey and chicken, respectively. Genus level coverage was 73% and 93%, respectively, and from mostly caecal sample materials. Both poultry birds also had unique microbiomes with similarity at the species level of up to 16% [19].

An analysis of turkey microbiome sequences revealed the identities of 69 bacteria genera [19]. Of these, 37 belonged to the Firmicutes with > 5% represented by *Lactobacillus, Clostridium* and *Ruminococcus* genera. Other sequences belonged to the *Blautia*, *Virgibacillus*, *Ethanoligenes*, *Eubacterium*, Clostridiales family XI incertae sedis, *Faecalibacterium*, *Bacillus*, *Butyricoccus* and *Megamonas* genera. As in chickens, Bacteroidetes such as *Paraprevotella* and *Prevotella* predominated. *Aeromonas* and *Desulfohalobium* predominated the Proteobacteria. While *Alkaliphillus*, *Pectinatus*, *Blautia*, *Eubacterium*, *Butyricoccus* and *Butyrivibrio* made up ≥ 1%, the *Faecalibacterium*, *Clostridium*, *Megamonas* and *Ruminococcus* genera belonging to the Firmicutes represented ≥ 5% of caecal sequences analyzed [19].

### The turkey enteric virome

Viruses such as astroviruses, rotaviruses and reoviruses have been found in the gut of healthy turkey birds [57]. Others such as reoviruses, papovavirus, enterovirus and coronavirus-like particles were observed in turkey intestines in connection with turkey blue comb disease [58]. Viruses such as coronavirus, calicivirus, reovirus, astrovirus, rotavirus, picornavirus, picobirnavirus, parvovirus and adenovirus have been detected in poultry enteric disease syndromes [59]. Infections and co-infections in turkeys are caused by a wide range of associated viruses which have a negative impact on their intestinal absorptive functions, thus impacting health and productivity [60].

### The goose and duck virome

The viral classes detected in goose using cloacal samples include the circovirus, avian coronavirus, Lake Sarah-associated circular virus-32 and Tunis virus. The analysis involved a new panel of viral primers and an NGS-based data module which were designed for efficient virus characterization [61]. An analysis of the Australian wild duck faecal samples using viral metagenomics showed the presence of 21 viruses which included the avian coronavirus and avian paramyxovirus [62]. Samples were prepared in such a way that allowed the elimination of host and elemental dexoyribonucleic acid (DNA) (from bacteria, parasites, fungi), and achieved maximal retention of viral nucleic acid for good NGS reads. Viral communities were structurally and functionally distinct and varied with environs and host habitats [63]. Research on poultry virome population, especially viruses that are not associated with poultry diseases but inherent within the gut environment is still emerging and is an aspect that is worth further exploration.

### Other members of the poultry microbiome

Little research data is available on the inherent fungal members (mycobiome) and other members of the poultry microbiome such as parasites, protozoa and archaea species. Most fungi reported are linked to diseases in poultry, but not listed as commensal species within the microbiome [64]. This is also observed with parasites such as *Histomonas meleagridis*, *Cryptosporidium parvum* and *Eimeria* species [65]. Only the methanogenic archaea, *Methanobrevibacter woesei* and related strains have been reported in chickens [66, 67]. Fungal species like the *Saccharomyces* may be found as part of the gut mycobiome and serve as an alternative to antibiotics. It is also frequently used in prebiotic and probiotic feed formulations for the enhancement of gut health. Fungal members of the microbiome make up a less abundant group (about 0.001% to 0.1%) compared to bacteria. They may also play integral functions in metabolism, immune modulation, and in balancing the microbiome structure and profile. Again, factors which affect the general microbiome structure may also affect the fungal population [12]. The utilization of 16S ribosomal RNA gene amplification of chicken caecal samples revealed the presence of a methanogenic archaea phyla which had a 99% similarity to *Methanobrevibacter woesei*. The same microorganism was identified in goose faeces [66].

As regards the poultry phageome, little is known of the healthy poultry gut phage community [68]. Classification of phages is done based on the nucleic acid present, morphology, their target bacteria or site and the phage life cycle (lytic or lysogenic). More than 90% of bacteriophages are tailed and belong to the Myoviridae, Siphoviridae and Podoviridae phage families within the order Caudovirales [68]. In a study by Day et al. [49], the presence of a Siphoviridae phage, *Propionibacterium* phage PA6, as well as the T4- and P2-like phages in the Myoviridae family were demonstrated in chicken broilers. Other phages detected include the positive, single stranded RNA phage in the Leviviridae family and Adenoviridae phages, which had been reported earlier in turkeys [59]. More recently, bacteriophages capable of ingesting *Campylobacter* bacteria were reported. The chicken caeca samples used were derived from large poultry farms which did not contain *Campylobacter* [69].

### Gastrointestinal tract functions and compositional variations in microbiome by anatomical sites

The poultry GIT is a complex environment made up of several families of bacteria, protozoa, fungi and viruses, but bacteria make up the dominant class [19]. When attached to the epithelium, the bacteria act as a protective barrier [70]. They produce vitamins (vitamins B and K), organic acids, bacteriostatic short chain fatty acids (SCFA) like acetic, propionic and butyric acids, antimicrobials (bacteriocins) and induce favourable immune reactions [70]. These metabolites derived from the gut microbiome have essential roles in enhancing metabolism, nutrient digestion and absorption for better poultry health, growth and wellbeing [9]. However, pathogens such as *Salmonella* and *Campylobacter* may also be found within the microbial community and pose major health risks to humans [71].

The poultry gut is specialized for nutrient digestion and absorption [13]. Sequencing approaches therefore give better insight into the taxonomic diversity of nutrient metabolising species that work together within the host. The crop, proventriculus and gizzard (stomach), duodenum, jejunum and ileum (small intestine), caeca, large intestine, colon and cloaca make up the poultry GIT [72]. Each part plays a different role which is linked with the microbiota dynamics. These roles are essential in study design and ascertaining sampling techniques [73]. The chicken caecal microbiota are involved in recycling of nitrogen from uric acid with the production of essential amino acids and non-starch polysaccharides (NSPs) digestion [74, 75]. The physiology of poultry digestion also affects faecal and colon microbiome profiles [72]. The colon does not retain much digesta and is short (about 10 cm) in adult chickens. Feed ingestion to excretion from the cloaca takes about 2 h [76]. The faecal and colon microbial profiles might vary if samples are obtained prior to caecum voiding, and vice versa. If some digesta pass from the ileum to the colon immediately after voiding caecal excretion may be a mixture of ileal and caecal microbiota [72]. This is the common origin of reported variations in poultry microbiome profiles [77]. The incidence and prevalence of microbial species differ in poultry anatomical sites all the way from the crop to the large intestine [77].

The role of the poultry gut microbiome ranges from gut development and immunity to nutrition and physiological functions [78]. The large intestine, especially the caecum is responsible for futher absorption of nutrients, microbial fermentation and detoxification of harmful substances [25]. Bacteria have many functional roles in nutrient assimilation from animal feed through energy release from dietary fiber [79]. These bacteria are involved in the production of useful metabolites which include antimicrobial compounds (e.g., bacteriocins). The bacterial community lowers triglyceride levels and induce non-pathogenic immune responses that can provide nutrition and protection for the host [70].

The bacterial phyla, Firmicutes and Proteobacteria, are considered the predominant phyla found in the crop, gizzard, small intestine and cecum [20]. *Lactobacillus* species are found throughout the intestinal tract [35]. The crop which is found in the upper segment of the GIT is responsible for fermentation, starch hydrolysis, storage of food and as an acidic barrier (pH ∼ 4.5) [80]. The crop consists predominantly of Gram-positive facultative anaerobic bacteria that are found attached to the epithelium and in concentrations of 10^8^ to 10^9^ cfu/g [81]. The low pH environment of the gizzard acts as a barrier in preventing bacteria from entering the distal part of the intestinal tract. The principal function of the gizzard is to grind food particles in an acidic environment (pH 2.6) [13]. Lactobacilli, enterococci, lactose-negative enterobacteria and coliform bacteria are found in the gizzard [81]. Digestion of carbohydrates are promoted by *Lactobacillus*, enterococci, coliforms, as well as yeasts. The remainder of carbohydrates are digested in the caeca after passing through the lower GIT [82].

Bacterial density in the duodenum is generally low due to short transit time, its low pH, and presence of pancreatic and bile secretions [81]. Duodenal bacterial profile consists of clostridia, streptococci, enterobacteria and lactobacilli [83]. A reduction in the activities of digestive enzymes and deconjugation of bile acids makes the environment in the distal portion of the small intestine more favourable for bacterial growth [81]. The predominant phylum and genera in the small intestine are the lactobacilli, anaerobic bacteria and *Bifidobacterium* (Table 1) [89]. Roto et al. [90] also reported the presence of *Enterococcus faecium* and *Pediococcus* spp. in the small intestine. A complex bacterial community is found in the poultry (chicken) caecum due to the longer digestive transit times [86]. Firmicutes (approximately 50%–90% of all taxa) [20], Bacteroidetes, Actinobacteria and Proteobacteria make up the dominant caecum phyla [35]. *Peptostreptococcus, Propionibacterium, Eubacterium, Prevotella, Bacteroides* and *Clostridium* are the major genera recovered from the caecum by culture-dependant approaches [36]. The *Blautia*, *Anaerostipes*, *Veillonella*, *Butyrivibrio*, *Megamonas*, *Lactobacillus*, *Hespellia*, *Roseburia*, *Faecalibacterium* and *Ethanoligenes* genera made up > 1% of the caecal bacteria sequences identified [91]. A study by Nordentoft et al. [92] established the predominance of *Faecalibacterium* and *Butyricimonas* compared to other genera. The observed variation was attributed to the sampling and analytic techniques used. Archaea are generally present in lower concentrations [66], but the predominant archaeal genus found in chicken caeca is the *Methanobrevibacter*. Other archaeal taxa may exist in the gut environment and be involved in fermentation with the release of methanogenic dissipation of hydrogen [66, 67].

**Table 1:** Parts of the poultry GIT, their functions and associated microbiota

Gut part	Popular microbiota	Function	Gene occupation in the gut, %	References
Crop	Actinobacteria, Firmicutes, *Bacteroides*, Proteobacteria.	Feed storage and pre-treatment	Na	[72, 84]
Gizzard	Firmicutes (*Lactobacillus*, *Enterococcus*), coliforms	Feed grinding, low pH acts as a microbial barrier	Na	[13]
Duodenum	Clostridia, Streptococci, Enterobacteria and Lactobacilli	Reception of digestive enzymes from the pancreatic and bile ducts, dilution of digesta by secreted bile	Na	[85]
Ileum	Proteobacteria, Firmicutes, *Cytophaga*, *Flexibacter*, *Bacteroides*, Actinobacteria and Cyanobacteria	Passage of small amounts of digesta to the caecum	Na	[78, 84]
Caecum	Firmicutes, Actinobacteria, *Bacteroides*, Proteobacteria, Clostridiales and Anaerobic microbes, plus *Campylobacter*, *Helicobacter* and *Megamonas*; Fusobacteria (*Fusobacterium* sp.), Elusimicrobia (*Elusimicrobium* sp.), Synergistetes (*Cloacibacillus* sp.), Spirochaetes (*Treponema* sp.) or Verrucomicrobia (*Akkermansia* sp.).	Nutrients fermentation; Polysaccharides to short chain volatile fatty acids (SCFA) using enzymes (carbohydrate esterase, polysaccharide lyase, and glycoside hydrolase); Host performance and health	20%	[35, 72, 74, 86–88]
		Protein and amino acids metabolism, Effective nitrogen metabolism	9% 1%	[13, 75]
		Fatty acid and lipid metabolism	1%–2%	[75]
	Methanogenic Archaea	Na	Na	[42, 71, 75, 86]
Large intestine	Firmicutes (*Lactobacillus*), Proteobacteria (*E. coli*)	Retention of little or no digesta	Na	[25]

*Na* Not available

### Microbiome engineering in poultry health and conservation

Improving poultry livestock health for conservation purposes may involve microbiome engineering (ME). ME can be achieved through various means to facilitate poultry health and conservation [93]. Since molecular techniques have become quite inexpensive and available globally, and microbiome toolkits expanded in the last two decades, microbiome research has increased, and scientific information on their role in health (metabolism, disease, immunity, nutrition, fitness, behaviour) and conservation continue to produce new and interesting outcomes [93]. The application of sequencing methods in poultry microbiome analysis could significantly expedite the acquisition of fascinating results of importance to both animal and human health and disease trends. ME implies that poultry systems can be subjected to experimental manipulations for the purpose of enhancing their health and achieving conservation [94]. In order to improve health and poultry products production, microbiome conservation has become a viable option, and this too given the rise in world population and demand for reduced antibiotic use in livestock. Targeting the microbiome for poultry conservation may also help fight infectious diseases and decrease morbidity and mortality rates in poultry [95]. ME is discussed under subheadings in the following paragraphs.

### Means of changing microbiota composition

#### Antibiotic utilization

Besides the beneficial use of antibiotics in poultry infections therapy, antibiotics may cause disruption in the presence of favorable microbial species [96], as well as other negative health issues in poultry animals [97]. A disturbed or disrupted microbiome impacts poultry heath. This is observed with the report of a different micrbiome profile in healthy animals compared to the profile of a dysbiotic animal gut [98]. In other words, antibiotic use can adversely affect poultry gut microbiome conservation if inappropriately used. However, besides the careful monitoring of their use in infections treatment, the unexpected outcomes may require a pause or stoppage in their use in stimulating poultry growth.

#### Diet supplementation

##### Probiotics

A viable possibility to enhance poultry health and restore dysbiosis (DB) involves the use of probiotics. Diet supplementation with live microbiota called probiotics bring about health benefits following appropriate use [99]. For example, some microorganisms can efficiently digest feed fibres, as well as other nutrients to make them readily available to the animal host [100]. Probiotic lactic acid bacteria (LAB), for example, *Lactobacillus* sp. can produce beneficial molecules (lactic acid) that are antimicrobial in nature during digestion or fermentation of substrates which are useful to the host. They achieve this through the modification or stabilization of the inherent microbiome or microbiome environment. Some LAB are gut commensals. Bacteriocins are antimicrobial moiety secreted from *Lactobacillus acidophilus* which can competitively prevent pathogen colonization and attachment (competitive exclusion) [101], thus reducing pathogenesis [102]. Probiotics are also able to change epithelial turnover and biofilm structures [103]. Probiotics as immunoregulators have come under investigation and require more research to understand the underlying mechanisms. Immune regulation in form of decrease in *Salmonella* and enteropathogenic *E. coli* (EPEC) population, and upregulated antibody secretion have been reported in chickens fed with *Lactobacillus* supplemented feed [104].

Other probiotic examples include yeasts like *Saccharomyces* sp. which restore the gastrointestinal microbiome by reducing acidosis risk common in ruminants, and balancing gut pH [105]. Also, *Acremonium charticola*, *Aspergillus awamori*, *A. niger*, *A. oryzae*, *Chrysonilia crassa* and *Rhizopus oligosporus* [64] are examples of filamentous fungi with probiotic potential. However, their potentials are yet to be tested in poultry [106]. In addition, broilers’ fitness has been improved with probiotics and probiotic supplemented feeds [107]. Improved egg quality [108], egg size and enhanced egg production have been achieved with *Enterococcus faecium* and *Bifidobacterium thermophilus* in layers [109]. Despite the benefits of probiotics, controlled research on probiotic strains, their culture and isolation, as well as *in vivo* assay for efficacy and mechanism(s) of action remain to be determined. Studies on specificity or broad effect of single or multi-strain probiotic mixtures across animal hosts are also pertinent. Such studies could be followed by the determination of action mechanism(s) which may be either parameter dependent (diversity, microbiome, temperature) or constitutive [101]. It may also be essential to consider the developmental stage of an animal prior to probiotic application and probiotic development [95].

##### Prebiotics and enzymes

Besides probiotics, prebiotic diet supplements may also be used to stimulate growth. They are substrates or nutrients that can improve the growth of beneficial species within the microbiome [110]. Microbial by-products such as enzymes could also be utilized as supplements to break down or ferment inaccessible substrates. For example, enzymes breakdown fibrous substrates, release trapped nutrients and make them available within the microbiome environment. The nutrient-rich environment contributes to the improvement of the microbiome and poultry health. As an immense advantage, the use of supplements in ME and DB restoration are generally regarded as safe (GRAS) and less regulated compared to antibiotics [111]. Poultry fitness had been enhanced directly using the prebiotic fructo-oligosaccharide (FOS), which was not possible with mannan-oligosaccharide (MOS). The FOS improved activity of beneficial species but did not impede pathogen activity [112]. In mildly stressed environments, MOS has been reported to increase the gut surface-area to volume ratio through increase of goblet cell numbers and villi height in poultry birds [13]. Prebiotic galacto-oligosaccharide (GOS) also influence the chicken gut flora by regulating the bird’s innate immunity (upregulated presence of interleukin-17A (IL-17A) over IL-10 in chicken caecum and ileum). GOS also influenced microbiome profile by increasing the relative abundance of *Lactobacillus johnsonii* in GOS-fed broilers compared to *Lactobacillus crispatus*, for improvement in broiler performance [113].

#### Faecal microbial transplantation (FMT)

A dysbiotic microbiome, as well as feed efficiency (FE) may be improved using FMT with transplant samples derived from a healthy poultry donor [114]. However, outcomes of FMT treatments are variable, thus limiting its efficacy as demonstrated in recent attempts [115, 116]. In poultry, however, the use of FMT with or without probiotics have protected against *Clostridium perfringens*, *Salmonella*, *Campylobacter*, *Listeria* spp. and *E. coli* pathogens. Such protection was probably achieved by competitive exclusion and environmental modifications through the secretion of various molecules [18]. A recent study established that the commensal chicken microbiome can be engineered to regulate natural and hereditary immune reactions against influenza H9N2 virus following chick treatment with *Lactobacillus* and FMT. Thus, the combination of a probiotic and FMT served to promote a healthy microbiome environment for enhanced defence against the influenza virus [96]. The usage of FMT in poultry remains a budding research area that could be explored to expand potential applications.

### Techniques for changing microbiome composition and environment

#### Selective targeting of microbiome species

This often involves the creation and rewiring of specific actors within a microbiome to perform desired tasks such as use of phages in phage therapy and microbial gene editing in the CRISPR-Cas gene editing system. Usually, the target microorganism is often integral in the microbial ecosystem and would have a ripple effect within the microbiome. Bacteriophages are used to target specific bacterial strains in a microbiome in a technique known as phage therapy. It may be applied in stopping unintended consequences in humans from consumption of poultry products infected with *E. coli*, *Listeria*, *Salmonella* or *Campylobacter* [117]. The delivery of target molecules across cell membranes or induction of apoptosis in target microbial cells have also been achieved with bacterial secretion systems [118]. Certain commensal poultry microbiome species may also be selected to serve as drug delivery vehicles or for the secretion of cytokines [118].

##### CRISPR-Cas9 and other systems

Specific genes in a microbe may also be chosen for editing using the CRISPR-Cas9 gene editing system [119]. The Cas9 has been successfully demonstrated for the gene-targeted production of mutant spermatozoa, which were useful in the generation of both homozygous and heterozygous mutant chicken offsprings [120]. Gene editing in combination with electroporation has been shown to affect the function of target genes in chick embryo somatic cells [121]. The system allows for the deletion of specific gene regions (virulence factor coding genes) or used as an antimicrobial via self-targeted removal of the resistant region [122]. Microbiomes may also be edited using pyocins which remove specific microbial strains in a microbiome by puncturing the cell membrane and killing targeted species [123]. Some added prospects worth exploring include engineered phages and bacteria as gene and protein transfer vehicles. Still, these potential applications are far from happening soon given the possible risks which need to be circumvented prior to use in poultry [124].

##### Phage therapy

Phages infect and replicate within bacteria but are selective of the bacteria they infect. Phage therapy impacts the microbiome by removing foodborne pathogens such as *E. coli* [125], *C. jejuni*, *L. monocytogenes*, *Staphylococcus aureus*, *Salmonella* spp., methiciliin-resistant *Staphylococcus aureus* (MRSA) [68], and *C. perfringens* [126]. Unlike antibiotics, phages are more specific in their target and aid in conservation of the commensal gut microbiome. Phage therapy is therefore a viable alternative to antibiotics and makes an excellent tool in poultry infections treatment [68].

### Poultry microbiome characterization and technological advancements

The poultry gut microbiota affects poultry health and growth through essential functions such as improvement in nutrient absorption and immune system strengthening and modulation [13]. Choi et al. [84] opined that for a detailed insight into poultry microbiome function and diversity, metagenomic tools may be used in the analysis of chicken gut microbiota with a view to taxonomically characterize and infer biome functions. The same analytic tools could be useful in proposing gene sets that could serve as indicators for poultry health. The authors also advocated that the chicken microbiota could be manipulated to enhance poultry wellbeing in the future [84]. Due to the gut microbiome complexity and diversity, the ability to fully comprehend the roles of the gut microbiome has been immensely impeded. To shed more light, metagenomic approaches are being used and continuously developed to help understand these roles, as well as aid in the ecological and nutritional predictive functions of the poultry gut microbiome. Studies that link feed type to microbiome profiles also require the application of metagenomic techniques [127]. Metaproteomic and 16S rRNA analysis have revealed that microorganisms identified from caecal samples belonging to the Carnobacteriaceae, Lachnospiraceae, Clostridiaceae, Erysipelotrichaceae, Streptococcaceae, Peptococcaceae, Ruminococcaceae, Lactobacillaceae, Anaeroplasmataceae, Succinovibrionaceae and Eubacteriaceae families have a relative abundance above 1% [127]. Metagenomic approaches into poultry microbiome investigate genetic information derived from specific host(s) or environments with the intention of determining their microbiome diversity and functions [128].

Prior to the emergence of metagenomic techniques, conventional methods for poultry microbiome studies such as the Sanger sequencing were used. It was however low-throughput and associated with insufficient genetic sequence data and high cost of sequencing. Fortunately, the high-throughput NGS platforms which were only recently developed, circumvent these shortfalls. NGS has paved the way for increased studies on livestock (including poultry) gut microbiome diversity and functions and continues to yield new and interesting genomic findings. It also sheds light on the roles of previously rare and unidentified members of the gut microbiome [128].

Elongation factors, ribosomal subunits are marker gene examples which are representative of microbial populations. In taxonomic resolution, the 16S rRNA gene coding region are more generally utilized to resolve microbiome compositions due to the hypervariable nature of the region [93]. For instance, there are nine highly variable regions in the bacterial 16S rRNA gene which are bordered by conserved regions. These regions are usually chosen as the primer sites for polymerase chain reaction (PCR). The differences in sequence of highly varied regions make accurate bacterial taxonomic classification possible when compared to 16S rRNA sequence databases like the National Centre for Biotechnology Information (NCBI) GenBank, Ribosomal Database Project (RDP) [129] and GreenGenes [130].

Some 16S rRNA gene bioinformatic pipelines have also been proposed for use with NGS-based microbiome profiling which suffice for derivation of taxonomic data through the processing of raw 16S rRNA sequence reads. Examples include, utilizing Illumina MiSeq, Ion PGM Systems and 454 pyrosequencing in 16S amplicon sequencing. The use of these pipelines would involve checks on chimeras and low-quality sequence reads [131], elimination of pyrosequencing-related read errors [132], and cluster production from similar sequences known as an operational taxonomic unit (OTUs) [129]. The most abundant sequences within similar OTUs which are considered representative sequences are thereafter compared to sequences within a database to determine taxonomic classification. The taxonomic data generated may also be useful in determination of degree of variance between sample types and microbiomes [93]. The OTUs also inform on microbiome diversity. Diversity has to do with how certain microbial species are evenly spread (symmetry or consistency) in a sample and is commonly termed the ‘alpha diversity’ or richness. Evenness and richness are integral indicators of livestock animal health [133]. A significantly reduced number of microbial species in a microbiome is indicative of low richness, while the presence of a few dominating taxa in samples point to low evenness and may suggest poor animal health. Qiime is one bioinformatic tool that can be used to obtain these alpha diversity indicators [134]. However, alpha diversity is thought to be a poor index for arriving at functional deductions for microbial species within a microbiome [135].

Alpha diversity only depicts a statistical synopsis of diversity in a single population [136]. Still, it has been reported that a gut microbiome demonstrating low diversity showed poor stability and health when compared with a highly diverse gut flora. In this way, alpha diversity can be related to basal inferences on microbiome functions and mechanisms [137]. However, when making comparison of the microbiome to ascertain shared taxa or OTU number and determining how microbial species functions vary across several microbiome, beta diversity is utilized. It is given in form of a similarity index (e.g., Bray-Curtis) which incorporates added information such as to what extent functions vary across several microbiome, as well as shared members between microbiome profiles of many communities [136]. Beta diversity derivation approaches may be qualitative (unweighted UniFrac) or quantitative (weighted UniFrac) [138]. Sequencing errors must be accounted for in microbial diversity analysis [139]. Such errors are circumvented with the use of high-throughput Illumina MiSeq and HiSeq sequencing platforms where errors are more easily managed computationally compared to sequencing errors generated from 454 pyrosequencing [140]. Chimeric sequences may also be generated from different 16S rRNA genes in the process of genetic amplification. They can however be removed using self-query sequences or reference sequences [131]. Quality checks involving a process of filtering sequence reads are key to ensuring microbial diversity analyses are accurate and optimal. Recent research efforts have also floated new primer panels and NGS-based module designs with significant efficiency for characterization of both old and new viral agents in mixed biological samples. These methods serve for both virome detection and identification, and diagnosis of viral diseases [61].

On the other hand, unlike the OTU approach, the Amplicon Sequence Variants (ASVs) method determines the exact number of times that target sequences were read. Sequences are then filtered using a threshold confidence level to generate exact sequences without the use of databases or clustering. This makes direct comparison with databases or studies utilizing similar target genes possible. The ASVs approach gives a higher resolution for precise information on diversity and identification to the species or strain level. ASVs are also known as ‘sub-OTUs’ [141].

### The poultry microbiome – health relationship

Poultry intestinal health is important for an efficient and sustainable GIT physiology [142]. The maintenance of a healthy gut is complex and relies on a fine balance between the immune system and the endogenous microbiota [35]. A healthy poultry gut is generally involved in maintaining intestinal homeostasis by a complex network of cells and their secreted soluble products [143]. The intestinal microbiota is involved in modulating host immune system, influences the normal structural and functional organ development, and host metabolism [144]. Mucosal immune responses to resident intestinal microbiota can distinguish commensal from pathogenic bacteria [79]. The repertoire of the T-cell receptors found in both the gut and the spleen are known to be affected by the diverse microbiota found in the avian GIT [145]. The gut microbiota is also involved in the modulation of B-cell response and immunoglobulin A (IgA) production. IgA plays an important role in regulating the composition of the gut microbiota by specifically binding to the bacterial epitopes [146].

The microbiota found in the poultry gut promotes the beneficial development of the intestinal mucus layer and epithelial monolayer, the exclusion of pathogenic microorganisms [147], polysaccharide degradation [75], and energy provision in the form of amino acids and SCFA [74]. Vitamins such as vitamin K, and water-soluble vitamin B such as biotin, cobalamin, folates, nicotinic acid, pantothenic acid, pyridoxine, riboflavin and thiamine are synthesized by microbial communities in the gut [148]. Any disruption in intestinal health can affect one to several systemic functions [149]. Some of the negative effects that can occur are dysregulation of adaptative immune cells, disturbances in microbial metabolism leading to the conversion of pathobionts to pathogens, enzymatic degradation of the intestinal mucus, decrease in fat digestibility, and the production of toxic amino acid catabolites [150].

The increased proliferation of microbial communities occurs when there is a higher availability of undigested nutrients present in the hindgut [151]. This leads to disruption in the equilibrium between the gut microbiome and host, creating metabolic, pathogenic or sterile inflammation [152]. A healthy gut optimizes digestibility, reduces nutrient excretion and mitigates ammonia (NH_3_) and other gas emissions within the poultry housing environment. These gases may pose an environmental and health risk [153]. Conditions such as optimal temperature, production phase, bird size and air currents in the poultry farm can affect the health and productivity of poultry birds [154]. External environmental stressors such as temperature variations, drafts, dryness or humidity, and internal stressors can alter the intake of feed and intestinal motility resulting in reduced digestion [155]. Good ventilation within the poultry house is also key to minimize condensation and litter moisture [156]. These stressors can be detrimental to the immunological systems of poultry whereby these birds now lack the ability to maintain their GIT microbiota and health [157].

## Conclusions

The shift from traditional techniques (culture-dependent) to the more recent and advanced metagenomic approaches (culture-independent) have expanded our knowledge of the poultry microbiome diversity, microbiome population dynamics, as well as microbiome functions in poultry metabolism and health. Advancements in bioinformatic tools remain essential to make headway in this budding area of scientific research into analysis of the poultry gut microbiota. It is also our submission that all participating members of the poultry microbiome including the archae, fungi and parasites still require in-depth analysis and elucidation. This is important because most studies are focused on bacterial profiling, few on fungi and even fewer on viral and other microbial classes [158]. Microbial databases would be significantly improved by increasing research of understudied domains and members of the microbiome [159]. The influence of internal and external environmental factors should also be given more consideration in microbiome study designs. ME techniques could also be further explored in poultry health and conservation. It is also highly likely that with the continued improvements in identification and characterization technologies, we can expect the discovery of new members of the poultry microbiome. In addition, greater insight into their role in poultry health, metabolism and conservation can also be expected.

## Data Availability

None.

## Abbreviations

GIT: Gastrointestinal tract
RNA: Ribonucleic acid
FAO: Food and Agricultural Organization
APEC: Avian pathogenic *Escherichia coli*
NGS: Next generation sequeincing
DNA: Dexoyribonucleic acid
SCFA: Short chain fatty acids
NSPs: Non-starch polysaccharides
CFU/G: Colony forming unit per gram
ME: Microbiome engineering
LAB: Lactic acid bacteria
DB: Dysbiosis
EPEC: Enteropathogenic *E. coli*
GRAS: Generally regarded as safe
FOS: Fructo-oligosaccharide
GOS: Galacto-oligosaccharide
MOS: Mannan-oligosaccharide
IL-17A: Interleukin-17A
FMT: Faecal microbial transplantation
FE: Feed efficiency
MRSA: Methicillin resistant *Staphylococcus aureus*
PCR: Polymerase chain reaction
NCBI: National Centre for Biotechnology Information
RDP: Ribosomal Database Project
OTU: Operational taxonomic unit
ASVs: Amplicon Sequence Variants
IgA: Immunoglobulin A
NH_3_: Ammonia
